# Supplementary material for: Sgk1 upregulation in hippocampus-projecting amygdala neurons underlies the delayed onset of PTSD-like avoidance behavior
Source: Nat Commun. 2026 Apr 1;17:4683. doi: 10.1038/s41467-026-71129-0 (PMC13201608; doi:10.1038/s41467-026-71129-0)
Supplement: Supplementary file 1 — Supplementary Information [file 41467_2026_71129_MOESM1_ESM.pdf]

Supplementary Information for

Sgk1 upregulation in hippocampus-projecting amygdala neurons underlies the delayed onset of PTSD-like avoidance behavior

Jia-Xin Zou[1,2,3#], Wei-Zhu Liu[3,4#], Ya-Qing Li[1,2,3], Yuan-Yuan Li[5], Wen-Jie You[3], Han-Qing Pan[3,5], Chun-Yan Wang[3,6], Wen-Hua Zhang[1,2,3,6*] and Bing-Xing Pan[1,2,3*]

[#]These authors contributed equally: Jia-Xin Zou, Wei-Zhu Liu

*Corresponding authors: Bing-Xing Pan and Wen-Hua Zhang

Email: panbingxing@ncu.edu.cn, whzhang@ncu.edu.cn

Supplementary Figures

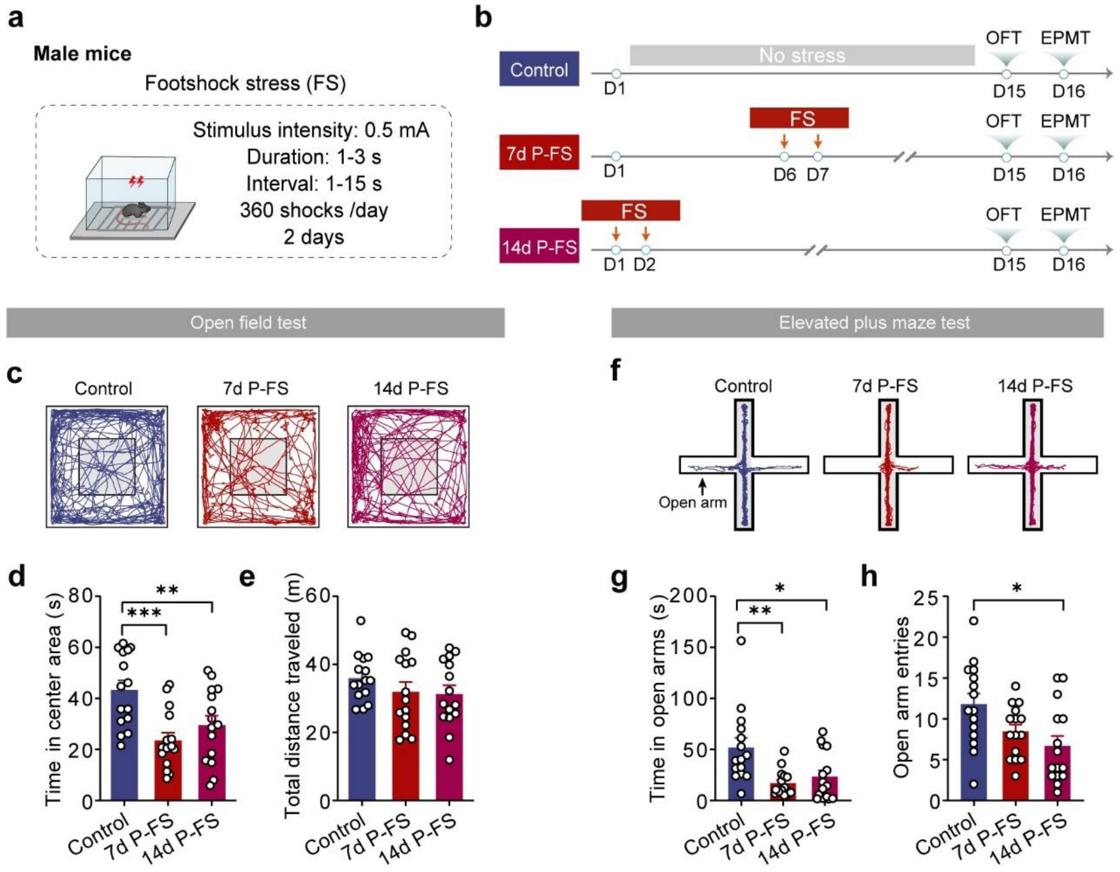

Supplementary Fig. 1 0.5 mA FS for 2 consecutive days causes enduring PTSD-like avoidance in male mice. a Schematic of FS procedure. **b** Time scheme for avoidance behavior test in the open field (OFT) and the elevated plus maze (EPMT). **c** Representative activity tracking in the OFT. **d**, **e** Time in center area (**d**) and total distance traveled (**e**) in OFT (n = 15 mice/group). **f** Representative activity tracking in the EPMT. **g**, **h** Time in open arms (**g**) and open arm entries (**h**) in EPMT, sample size as in panel (**d**, **e**). Data were analyzed using one-way ANOVA (**d**, **e**) and Kruskal-Wallis test (**g**, **h**), followed by Bonferroni-corrected, two-tailed pairwise comparisons for (**d**, **g**, **h**). Data were shown as mean ± SEM. *$p < 0.05$, **$p < 0.01$, ***$p < 0.001$. See Supplementary Data 1 for full statistical information. Source data are provided as a Source Data file.

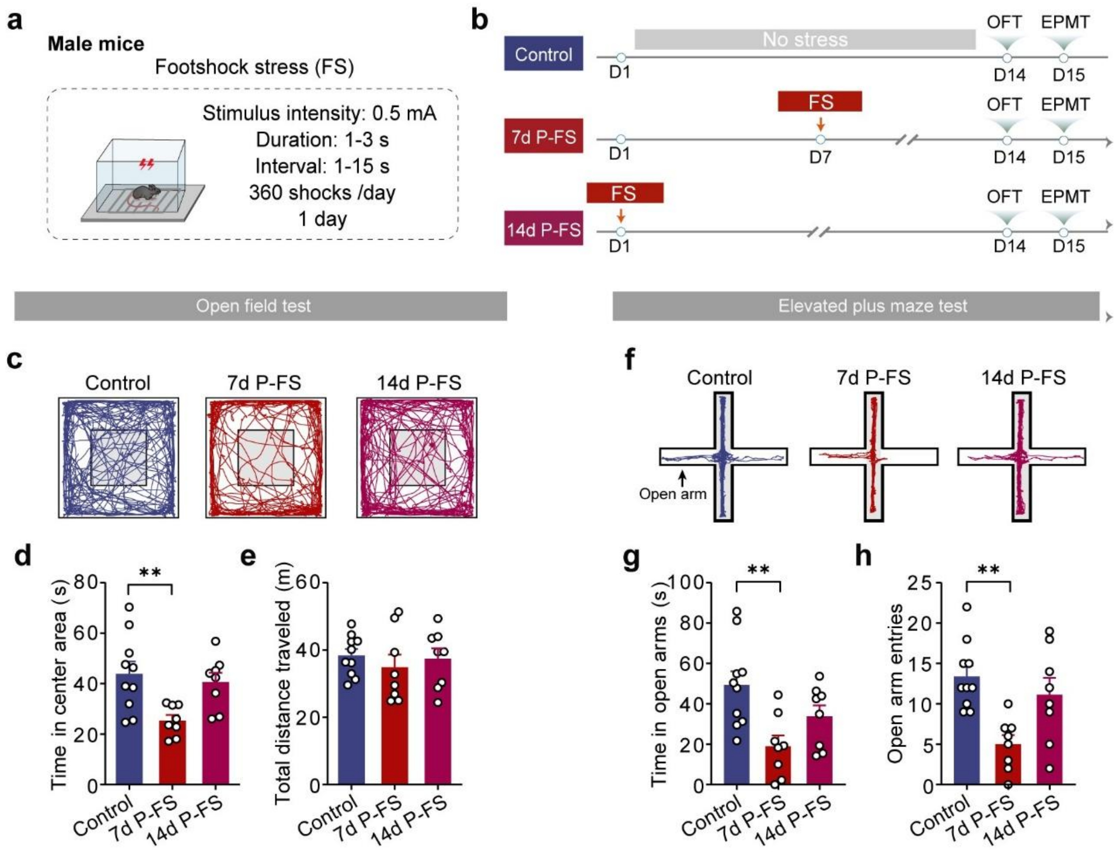

Supplementary Fig. 2 0.5 mA FS for a single day causes short-lasting PTSD-like avoidance in male mice. a Schematic of FS procedure. **b** Time scheme for avoidance behavior test in the open field (OFT) and the elevated plus maze (EPMT). **c** Representative activity tracking in the OFT. **d, e** Time in center area (**d**) and total distance traveled (**e**) in OFT (n = 8-10 mice). **f** Representative activity tracking in the EPMT. **g**, **h** Time in open arms (**g**) and open arm entries (**h**) in EPMT, sample size as in panel (**d**, **e**). Data were analyzed using one-way ANOVA (**d**, **e**, **g**, **h**), followed by Bonferroni-corrected, two-tailed pairwise comparisons for (**d**, **g**, **h**). Data were shown as mean ± SEM. $^{**}p < 0.01$. See Supplementary Data 1 for full statistical information. Source data are provided as a Source Data file.

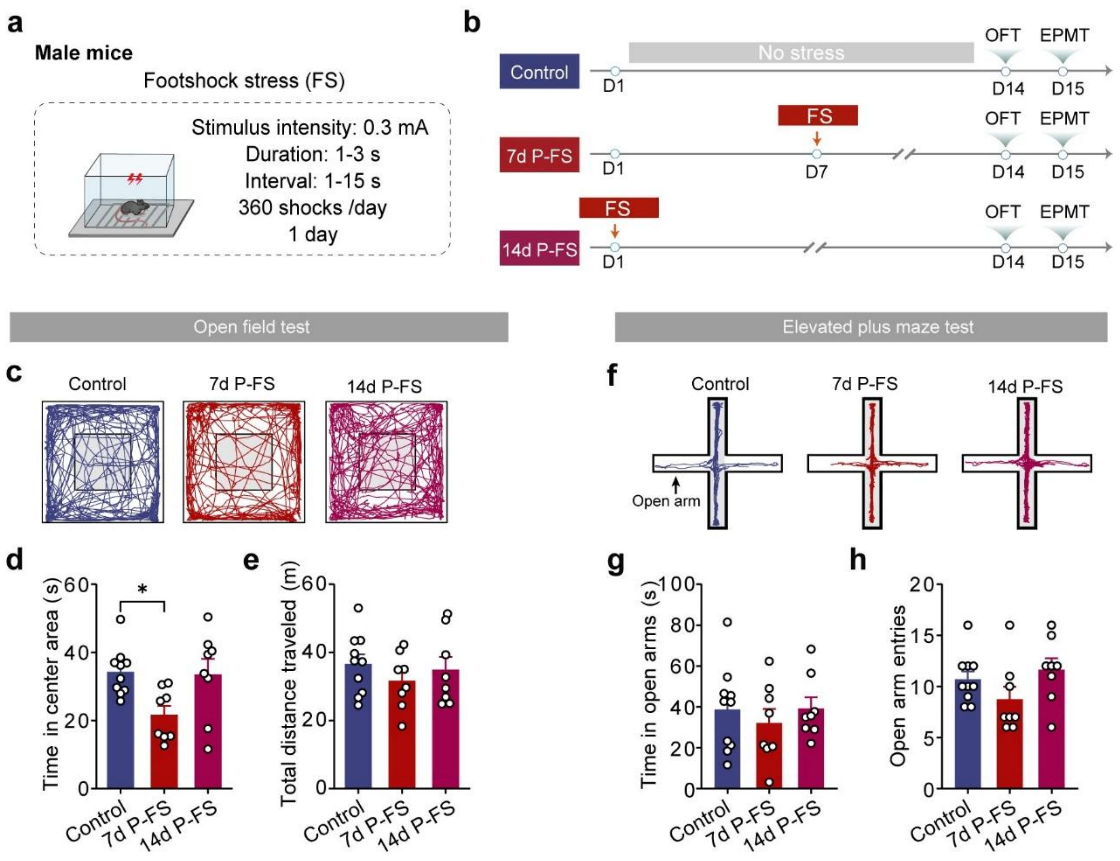

Supplementary Fig. 3 0.3 mA FS for a single day has little effect on PTSD-like avoidance behavior in male mice. a Schematic of procedure. **b** Time scheme for avoidance behavior test in the open field (OFT) and the elevated plus maze (EPMT). **c** Representative activity tracking in the OFT. **d**, **e** Time in center area (**d**) and total distance traveled (**e**) in OFT (n = 8-10 mice). **f** Representative activity tracking in the EPMT. **g**, **h** Time in open arms (**g**) and open arm entries (**h**) in EPMT, sample size as in panel (**d**, **e**). Data were analyzed using one-way ANOVA (**d**, **e**) and Kruskal-Wallis test (**g**, **h**), followed by Bonferroni-corrected, two-tailed pairwise comparisons for (**d**). Data were shown as mean ± SEM. $*p < 0.05$. See Supplementary Data 1 for full statistical information. Source data are provided as a Source Data file.

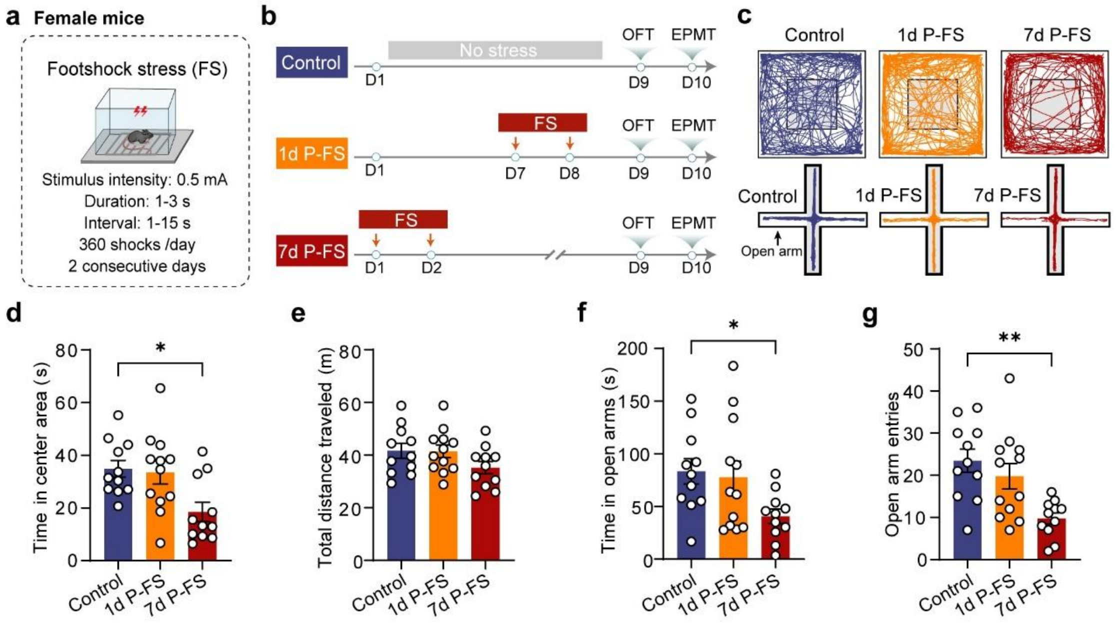

Supplementary Fig. 4 Footshock stress causes delayed PTSD-like avoidance behavior in female mice. a Schematic of FS procedure. **b** Time scheme for avoidance behavior test in the open field (OFT) and the elevated plus maze (EPMT). **c** Representative activity tracking in the OFT and the EPMT. **d**, **e** Time in center area (**d**) and total distance traveled (**e**) in OFT (*n* = 11-12 mice). **f**, **g** Time in open arms (**f**) and open arm entries (**g**) in EPMT, sample size as in panel (**d**, **e**). Data were analyzed using one-way ANOVA (**d**, **e**, **f**) and Kruskal-Wallis test (**g**), followed by Bonferroni-corrected, two-tailed pairwise comparisons for (**d**, **f**, **g**). Data were shown as mean ± SEM. $*p < 0.05$, $***p < 0.001$. See Supplementary Data 1 for full statistical information. Source data are provided as a Source Data file.

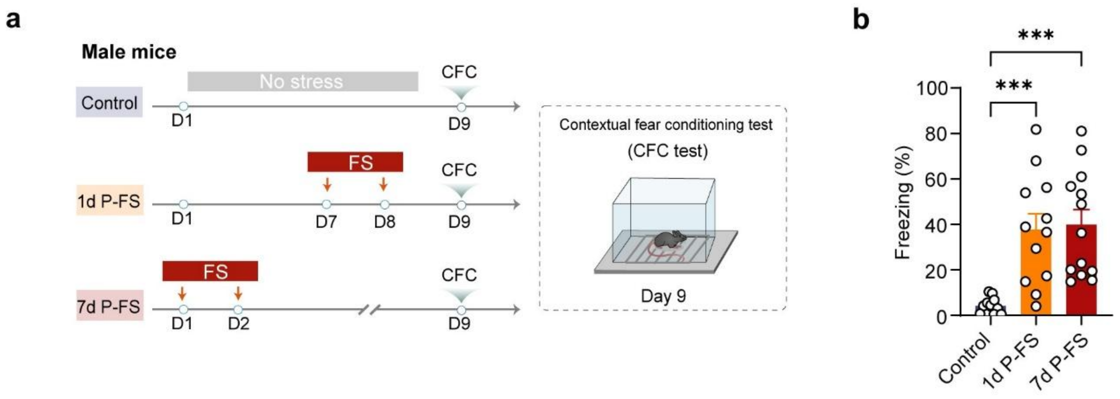

Supplementary Fig. 5 Footshock stress causes elevated fear memory in male mice. a Time scheme for contextual fear conditioning (CFC) test. **b** Summary plots of freezing level of mice in CFC test (n = 12-13 mice). Data were analyzed using Kruskal-Wallis test (**b**), followed by Bonferroni-corrected, two-tailed pairwise comparisons. Data were shown as mean ± SEM. ***$p < 0.001$. See Supplementary Data 1 for full statistical information. Source data are provided as a Source Data file.

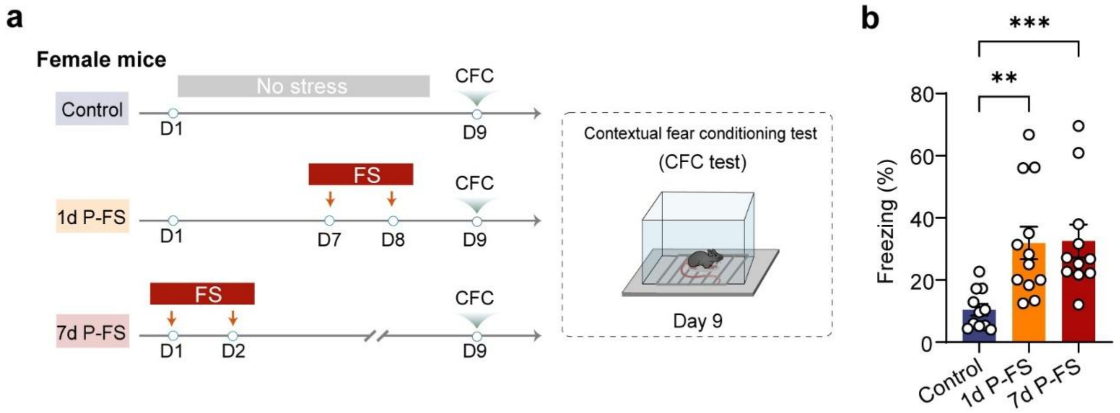

Supplementary Fig. 6 Footshock stress causes elevated fear memory in female mice. a Time scheme for contextual fear conditioning (CFC) test. **b** Summary plots of freezing level of mice in CFC test (n = 11-12 mice). Data were analyzed using Kruskal-Wallis test (**b**), followed by Bonferroni-corrected, two-tailed pairwise comparisons. Data were shown as mean ± SEM. $^{**}p < 0.01$, $^{***}p < 0.001$. See Supplementary Data 1 for full statistical information. Source data are provided as a Source Data file.

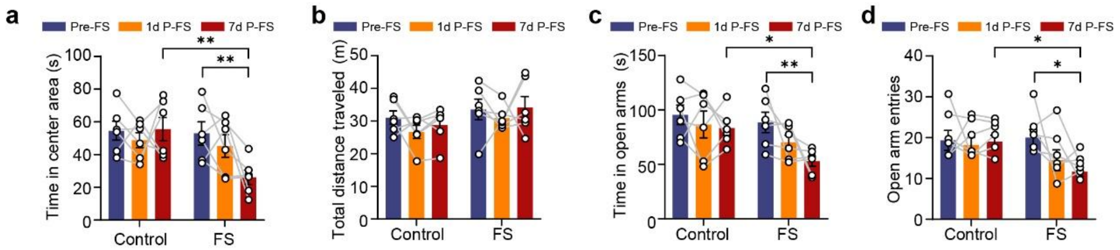

Supplementary Fig. 7 FS induces delayed PTSD-like avoidance behavior in fiber photometry recording male mice. **a**, **b** Time in center area (**a**) and total distance traveled (**b**) in OFT (n = 6 mice/group). **c**, **d** Time in open arms (**c**) and open arm entries (**d**) in EPMT, sample size as in panel (**a**, **b**). Data were analyzed using two-way RM ANOVA (**a**-**d**), followed by Bonferroni-corrected, two-tailed pairwise comparisons for (**a**, **c**, **d**). Data were shown as mean ± SEM. $*p < 0.05$, $**p < 0.01$. See Supplementary Data 1 for full statistical information. Source data are provided as a Source Data file.

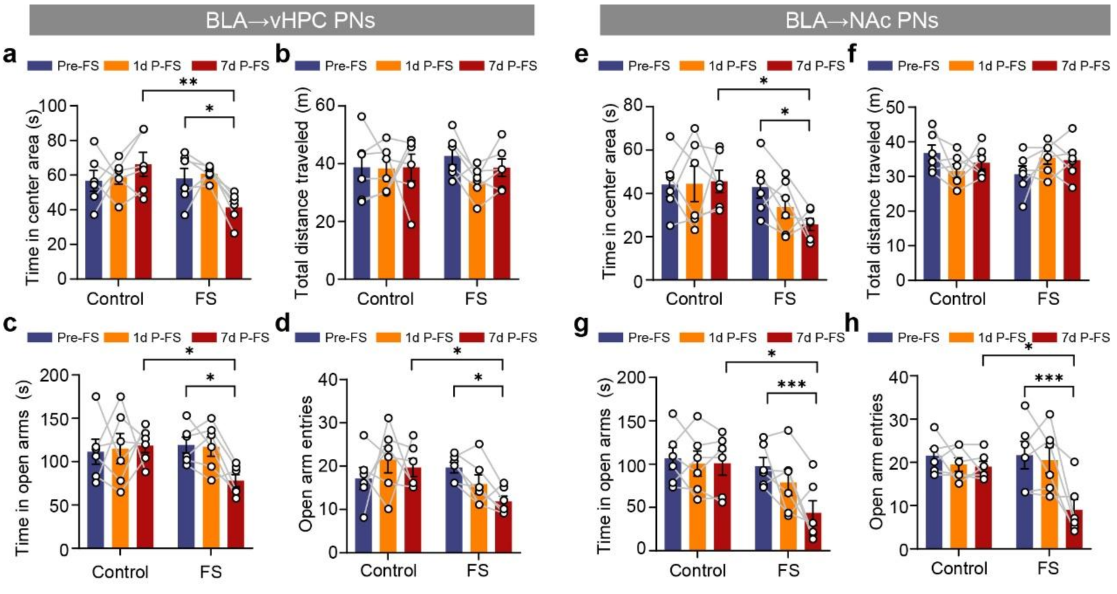

Supplementary Fig. 8 FS-exposed mice show delayed PTSD-like avoidance behavior at 7 days post-stress during fiber photometry recording. a, **b** Time in center area (**a**) and total distance traveled (**b**) in OFT from mice with specifically labeled BLA→vHPC PNs (*n* = 6 mice/group) **c**, **d** Time in open arms (**c**) and open arm entries (**d**) in EPMT from mice with specifically labeled BLA→vHPC PNs, sample size as in panel (**a**, **b**). **e**, **f** Time in center area (**e**) and total distance traveled (**f**) in OFT from mice with specifically labeled BLA→NAc PNs (*n* = 6 mice/group). **g**, **h** Time in open arms (**g**) and open arm entries (**h**) in EPMT from mice with specifically labeled BLA→NAc PNs, sample size as in panel (**e**, **f**). Data were analyzed using two-way RM ANOVA (**a**-**h**), followed by Bonferroni-corrected, two-tailed pairwise comparisons for (**a**, **c**-**e**, **g**, **h**). Data were shown as mean ± SEM. *$p < 0.05$, **$p < 0.01$, ***$p < 0.001$. See Supplementary Data 1 for full statistical information. Source data are provided as a Source Data file.

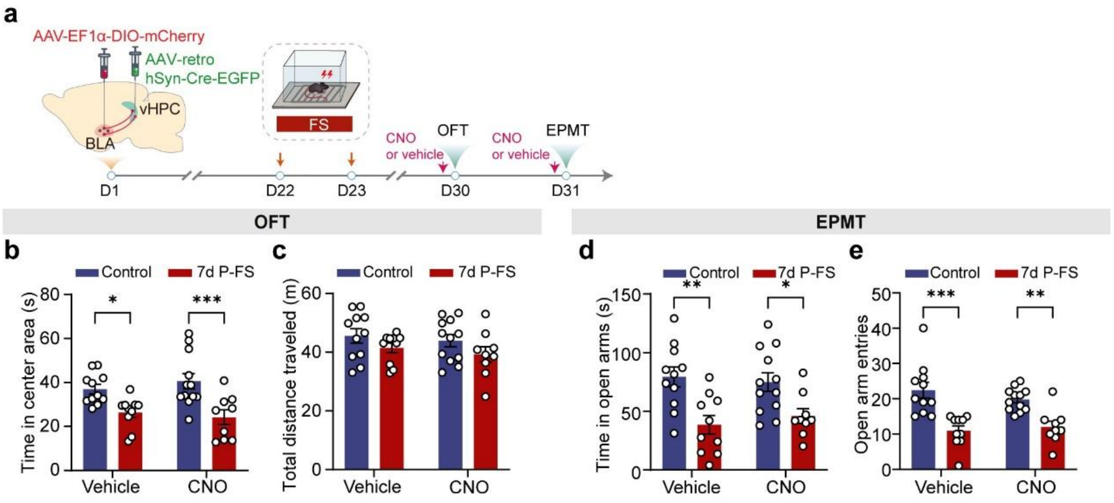

Supplementary Fig. 9 CNO produced no behavioral changes in mice expressing mCherry-only in BLA→vHPC PNs. **a** Schematic of the experimental procedures. **b**, **c** Time in center area (**b**) and total distance traveled (**c**) in OFT (*n* = 9-12 mice). **d**, **e** Time in open arms (**d**) and open arm entries (**e**) in EPMT, sample size as in panel (**b**, **c**). Data were analyzed using two-way ANOVA (**b**-**e**), followed by Bonferroni-corrected, two-tailed pairwise comparisons for (**b**, **d**, **e**). Data were shown as mean ± SEM. *$p < 0.05$, **$p < 0.01$, ***$p < 0.001$. See Supplementary Data 1 for full statistical information. Source data are provided as a Source Data file.

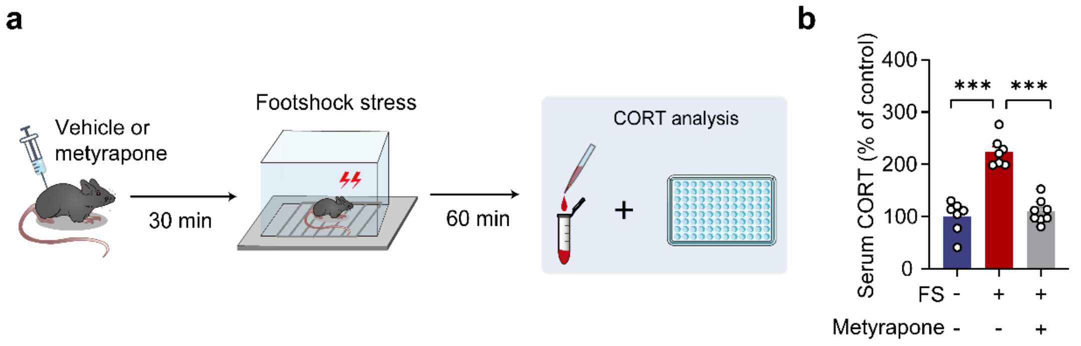

Supplementary Fig. 10 Pre-treatment with metyrapone prevents the rapid and transient rise in serum CORT levels caused by FS in mice a Schematic of experimental procedures. **b** Summary plots of normalized serum CORT level in vehicle+unstressed control, vehicle+FS and metyrapone+FS mice (n = 7-8 mice). Data were analyzed using one-way ANOVA (**b**), followed by Bonferroni-corrected, two-tailed pairwise comparisons. Data were shown as mean ± SEM. ***$p < 0.001$. See Supplementary Data 1 for full statistical information. Source data are provided as a Source Data file.

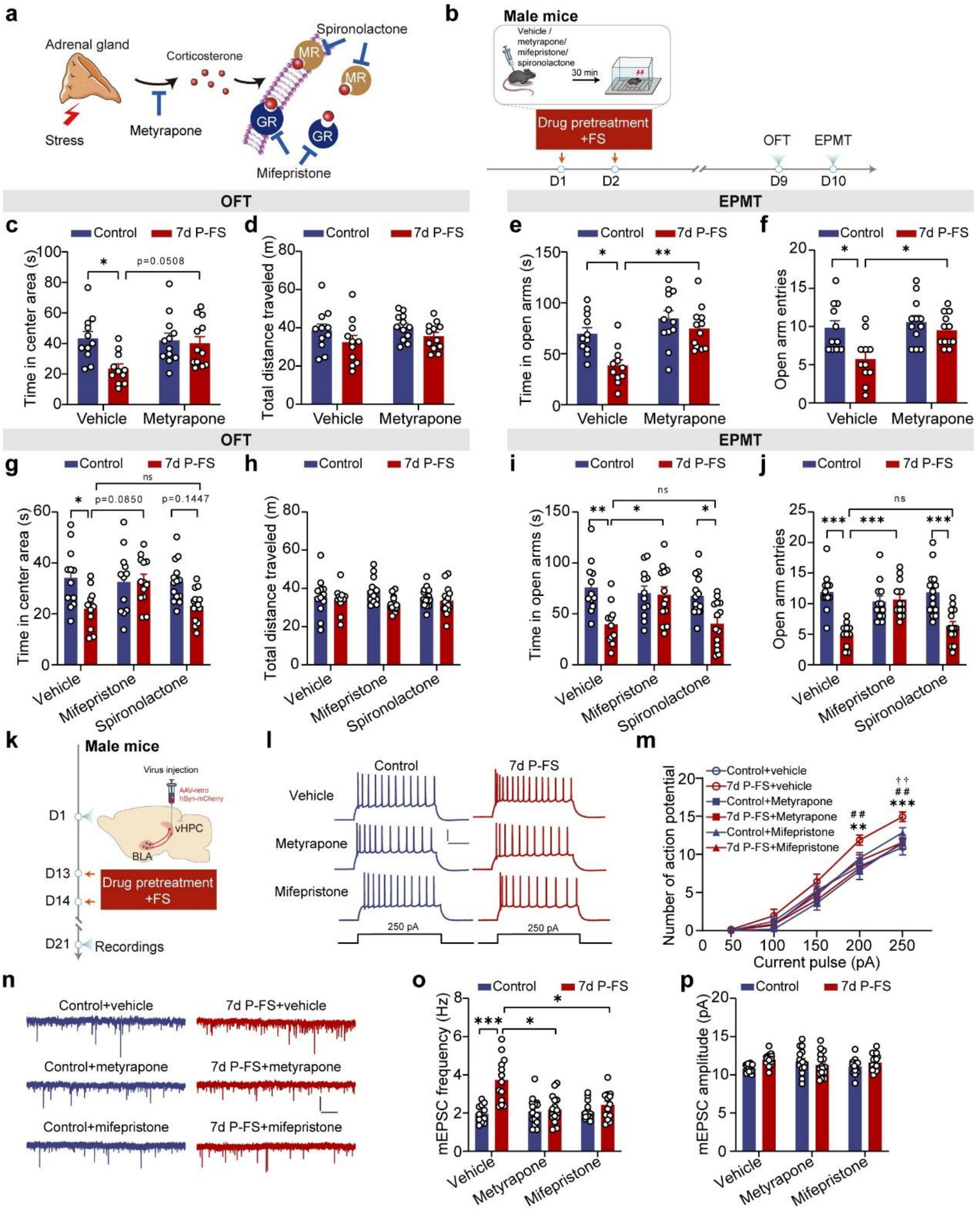

Supplementary Fig. 11 Blocking of CORT-GR signaling mitigates FS-induced increase in avoidance behavior and BLA→vHPC PNs overactivation in male mice. a Schematic diagram showing the inhibition of metyrapone, mifepristone and spironolactone on the CORT signaling. **b** Schematic of experiment procedures. **c**, **d** Time in center area (**c**) and total distance traveled (**d**) in OFT (*n* = 11-12 mice). **e**, **f** Time in open arms (**e**) and open arm entries (**f**) in EPMT, sample size as in panel (**c**, **d**). **g**, **h** Time in center area (**g**) and total distance traveled (**h**) in OFT (*n* = 11-14 mice). **i, j** Time in open arms (**i**) and open arm entries (**j**) in EPMT, sample size as in panel (**g**, **h**). **k** Schematic of experiment

procedures. **l** Representative firing traces (250 pA current injection). Scale bar: 200 ms, 30 mV. **m** Action potential number in response to varying current injection (n = 12-15 neurons/4 mice). **n** Representative mEPSC traces of BLA→vHPC PNs. Scale bar: 1 s, 20 pA. **o**, **p** Averaged mEPSC frequency (**o**) and amplitude (**p**) of BLA→vHPC PNs (n = 12-16 neurons/3-4 mice). Data were analyzed using two-way ANOVA (**c**, **d**, **e**, **g-j**), Kruskal-Wallis test (**f**, **o**, **p**) and three-way RM ANOVA (**m**), followed by Bonferroni-corrected, two-tailed pairwise comparisons for (**c**, **e-g**, **i**, **j**, **m**, **o**). Data were shown as mean ± SEM. ns, not significant. *$p < 0.05$, **$p < 0.01$, ***$p < 0.001$, ##$p < 0.01$, ††$p < 0.01$. See Supplementary Data 1 for full statistical information. Source data are provided as a Source Data file.

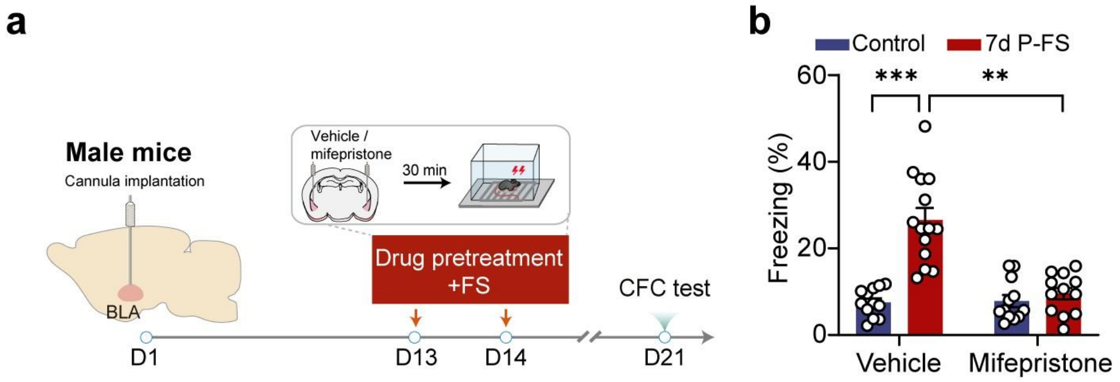

Supplementary Fig. 12 Blocking of GR signaling within BLA mitigates FS-induced increase in fear memory in male mice. a Schematic of experiment procedures. **b** Summary plots of freezing level of mice in CFC test (n = 12-14 mice). Data were analyzed using Kruskal-Wallis test (**b**), followed by Bonferroni-corrected, two-tailed pairwise comparisons. Data were shown as mean ± SEM. $^{**}p < 0.01$, $^{***}p < 0.001$. See Supplementary Data 1 for full statistical information. Source data are provided as a Source Data file.

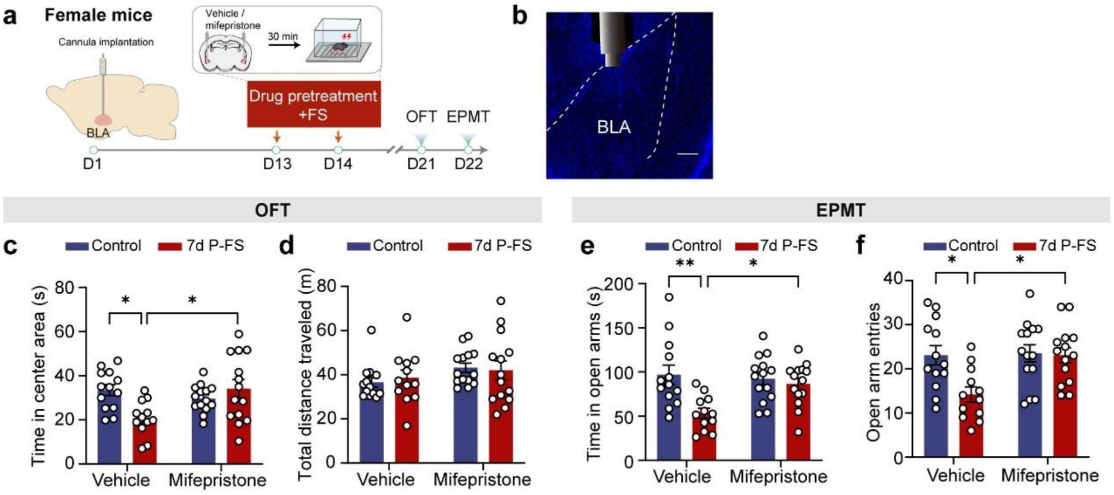

Supplementary Fig. 13 Blocking of GR signaling within BLA mitigates FS-induced increase in avoidance behavior in female mice. **a** Schematic of experiment procedures. **b** Representative images showing the cannula implantation in BLA. **c**, **d** Time in center area (**c**) and total distance traveled (**d**) in OFT (n = 12-14 mice). **e**, **f** Time in open arms (**e**) and open arm entries (**f**) in EPMT, sample size as in panel (**c**, **d**). Data were analyzed using Kruskal-Wallis test (**c**, **d**) and two-way ANOVA (**e**, **f**), followed by Bonferroni-corrected, two-tailed pairwise comparisons for (**c**, **e**, **f**). Data were shown as mean ± SEM. $*p < 0.05$, $**p < 0.01$. See Supplementary Data 1 for full statistical information. Source data are provided as a Source Data file.

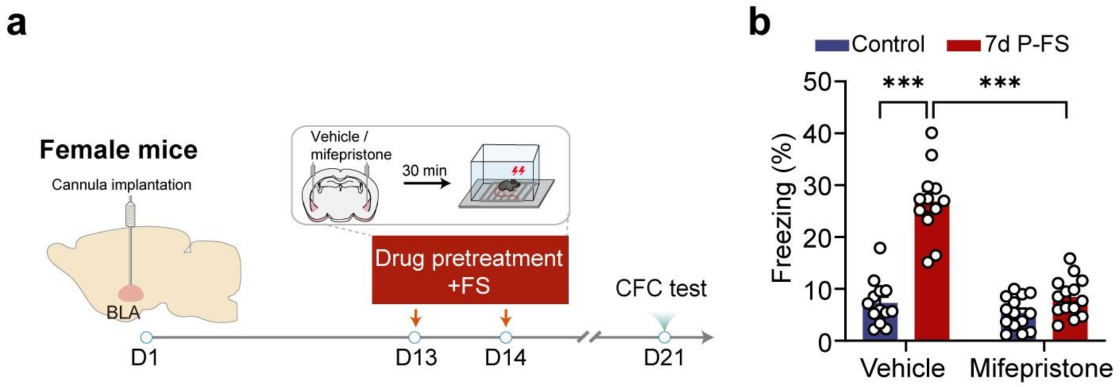

Supplementary Fig. 14 Blocking of GR signaling within BLA mitigates FS-induced increase in fear memory in female mice. a Schematic of experiment procedures. **b** Summary plots of freezing level of mice in CFC test (*n* = 12-14 mice). Data were analyzed using two-way ANOVA (**b**), followed by Bonferroni-corrected, two-tailed pairwise comparisons. Data were shown as mean ± SEM. *$p < 0.05$, **$p < 0.01$, ***$p < 0.001$. See Supplementary Data 1 for full statistical information. Source data are provided as a Source Data file.

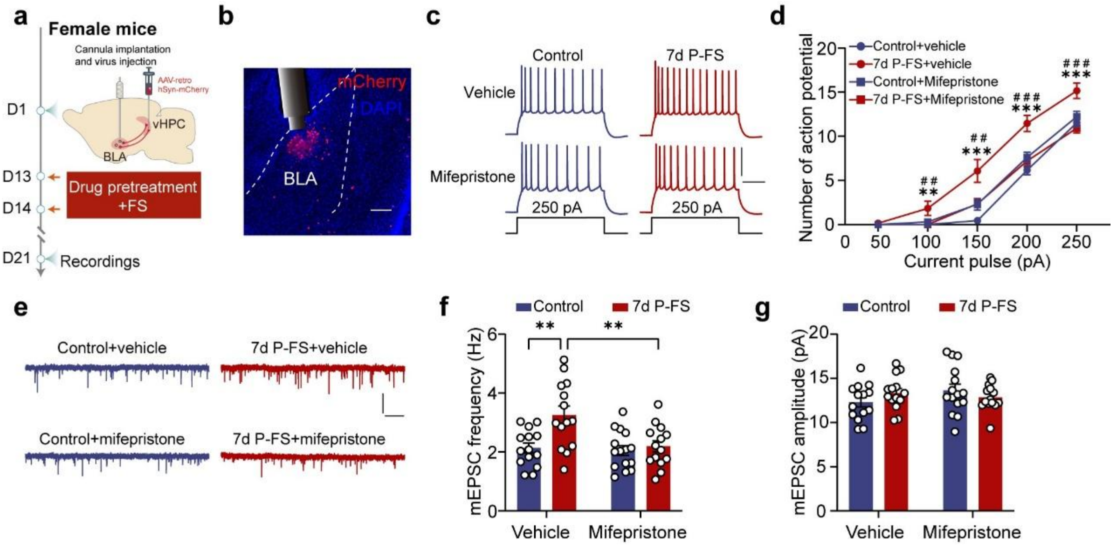

Supplementary Fig. 15 Blocking of GR signaling in BLA mitigates FS-induced BLA→vHPC PNs overactivation in female mice. a Schematic of experiment procedures. **b** Representative images showing mCherry expression in BLA→vHPC PNs and the cannula implantation in BLA. (n = 16 mice). **c** Representative firing traces (250 pA current injection). Scale bar: 200 ms, 50 mV. **d** Action potential number in response to varying current injection (n = 13-14 neurons/4 mice). **e** Representative mEPSC traces of BLA→vHPC PNs. Scale bar: 1 s, 20 pA. **f**, **g** Averaged mEPSC frequency (**f**) and amplitude (**g**) of BLA→vHPC PNs (n = 14-15 neurons/4 mice). Data were analyzed using three-way RM ANOVA (**d**) and two-way ANOVA (**f**, **g**), followed by Bonferroni-corrected, two-tailed pairwise comparisons for (**d**, **f**). Data were shown as mean ± SEM. *$p < 0.05$, **$p < 0.01$, ***$p < 0.001$, ##$p < 0.01$, ###$p < 0.001$. See Supplementary Data 1 for full statistical information. Source data are provided as a Source Data file.

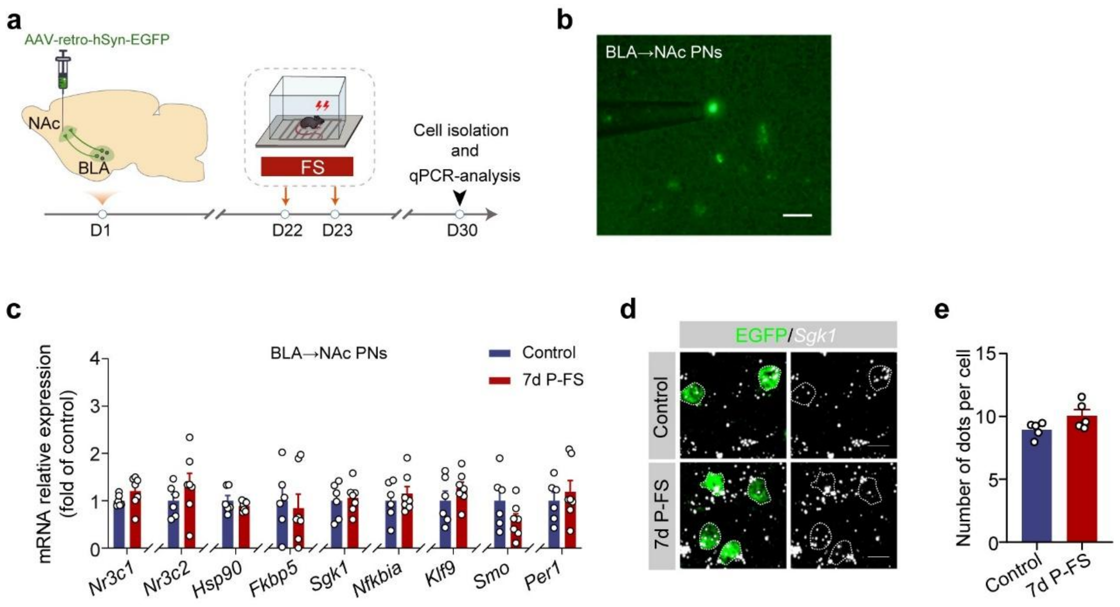

Supplementary Fig. 16 The impact of FS on CORT-related genes in BLA→NAc PNs. a Schematic of experiment procedures. **b** Representative image showing single-cell capture of BLA→NAc PNs. Scale bar: 50 µm. (*n* = 13 mice). **c** RT-qPCR analysis of genes involved in CORT signaling in BLA→NAc PNs (*n* = 6-7 mice). **d** Representative images showing EGFP expression and RNAscope for *Sgk1* transcripts (white dashed lines outline BLA→NAc PNs). Scale bar: 50 µm. **e** Averaged *Sgk1* transcript dots per cell (n = 5 mice/group). Data were analyzed using two-tailed unpaired *t* test (**c**: *Nr3c1, Nr3c2, Hsp90, Sgk1, Nfkbia, Klf9, Smo*; **e**) and two-tailed Mann-Whitney *U* test (**c**: *Fkbp5, Per1*). Data were shown as mean ± SEM. See Supplementary Data 1 for full statistical information. Source data are provided as a Source Data file.

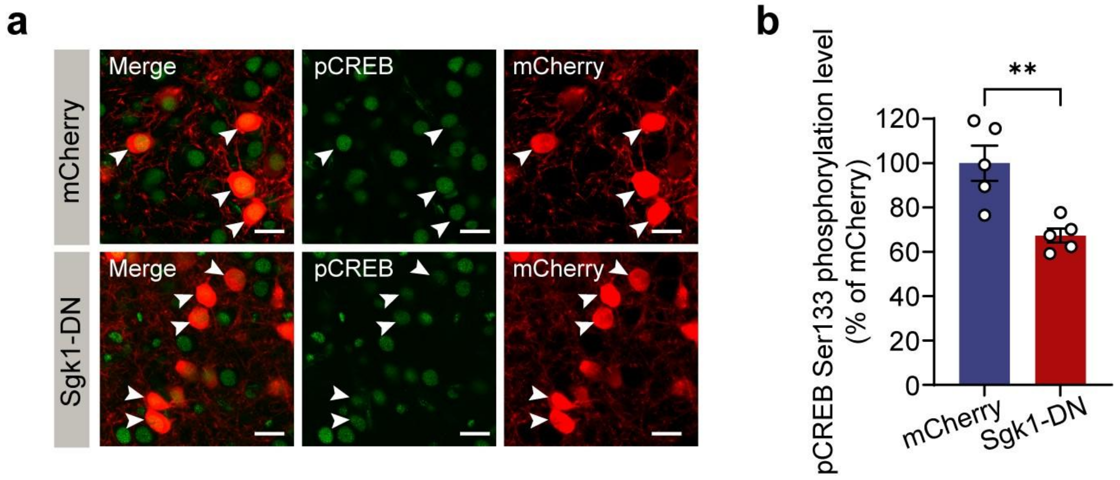

Supplementary Fig. 17 Overexpression of Sgk1(S422A) in BLA→vHPC PNs decreased pCREB levels in these neurons. **a** Representative images showing expression of Sgk1(S422A)-mCherry and immunostaining against pCREB in BLA neurons. Scale bar: 25 μm. **b** Summary plots showing pCREB expression in BLA→vHPC PNs (n = 5 mice/group). Data were analyzed using two-tailed unpaired t test (**b**). Data were shown as mean ± SEM. $^{**}p < 0.01$. See Supplementary Data 1 for full statistical information. Source data are provided as a Source Data file.

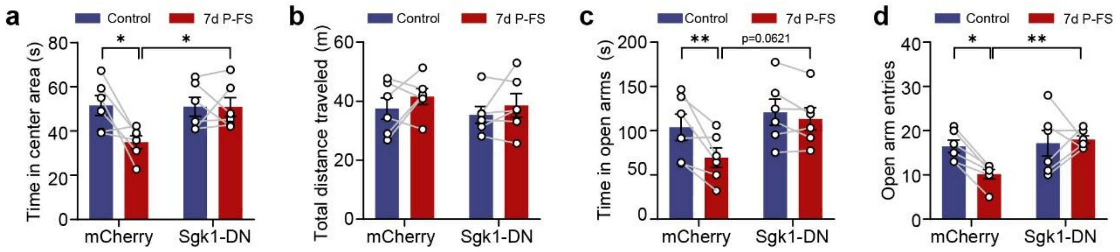

Supplementary Fig. 18 Sgk1-DN expression prevents the delayed PTSD-like avoidance behavior by FS in fiber photometry recording male mice. a, **b** Time in center area (**a**) and total distance traveled (**b**) in OFT (*n* = 6 mice/group). **c**, **d** Time in open arms (**c**) and open arm entries (**d**) in EPMT, sample size as in panel (**a**, **b**). Data were analyzed using two-way RM ANOVA (**a-d**), followed by Bonferroni-corrected, two-tailed pairwise comparisons for (**a**, **c**, **d**). Data were shown as mean ± SEM. *$p < 0.05$, **$p < 0.01$. See Supplementary Data 1 for full statistical information. Source data are provided as a Source Data file.

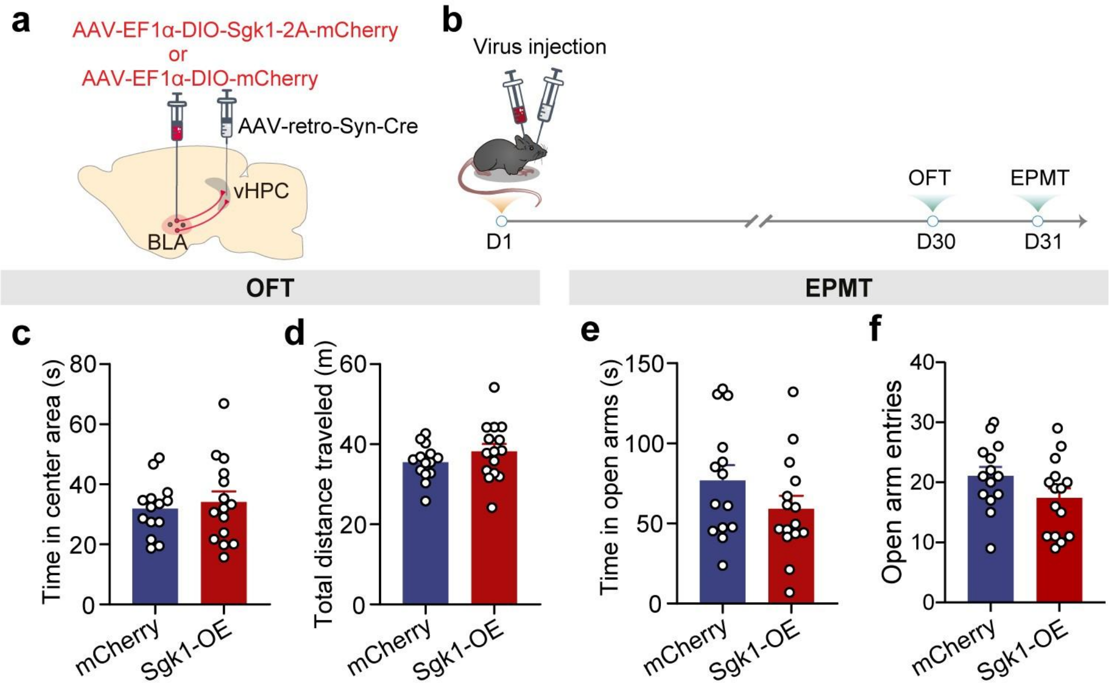

Supplementary Fig. 19 Sgk1 overexpression in BLA→vHPC PNs does not affect avoidance behavior in mice. a Schematic showing injection of rAAV-retro-Syn-Cre into vHPC and AAV-EF1α-DIO-Sgk1-mCherry (Sgk1-OE) or AAV-EF1α-DIO-mCherry (mCherry) into BLA. **b** Schematic of experimental procedures. **c**, **d** Time in center area (**c**) and total distance traveled (**d**) in OFT (n = 14-15 mice). **e**, **f** Time in open arms (**e**) and open arm entries (**f**) in EPMT, sample size as in panel (**c**, **d**). Data were analyzed using two-tailed unpaired t test (**c-f**). Data were shown as mean ± SEM. See Supplementary Data 1 for full statistical information. Source data are provided as a Source Data file.

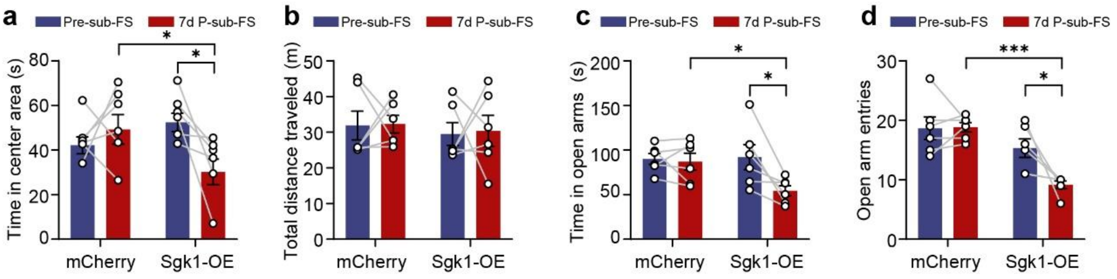

Supplementary Fig. 20 Sub-FS induces delayed PTSD-like avoidance behavior in Sgk1-overexpressing mice in fiber photometry recording. **a**, **b** Time in center area (**a**) and total distance traveled (**b**) in OFT (n = 6 mice/group). **c**, **d** Time in open arms (**c**) and open arm entries (**d**) in EPMT, sample size as in panel (**a**, **b**). Data were analyzed using two-way RM ANOVA (**a-d**), followed by Bonferroni-corrected, two-tailed pairwise comparisons for (**a**, **c**, **d**). Data were shown as mean ± SEM. *$p < 0.05$, ***$p < 0.001$. See Supplementary Data 1 for full statistical information. Source data are provided as a Source Data file.

Supplementary Tables

Supplementary Table 1. Membrane and action potential properties of BLA→vHPC PNs

**Membrane properties**	Unstressed control (n = 15)	1d P-FS (n = 13)	7d P-FS (n = 16)	$p_{\text{treatment}}$	$p_{\text{1d P-FS vs. Unstressed control}}$	$p_{\text{7d P-FS vs. Unstressed control}}$
RMP (mV)	-64.85 ± 0.81	-66.05 ± 0.92	-63.81 ± 0.66	0.1533	0.5982	0.6954
Input resistance (MΩ)	123.25 ± 6.88	130.27 ± 6.98	154.48 ± 8.54	0.0128	>0.9999	0.0099
AP threshold (mV)	-30.07 ± 0.73	-29.20 ± 0.91	-29.59 ± 0.67	0.7303	0.8672	> 0.9999
Half width of AP (ms)	1.40 ± 0.08	1.42 ±0.02	1.37 ± 0.05	0.2368	0.4060	>0.9999
Amplitude of AP (mV)	77.99 ± 2.70	78.16 ± 1. 63	79.37 ± 2.51	0.8983	>0.9999	>0.9999
fAHP (mV)	-6.29 ± 0.57	-6.69 ± 0.63	-6.40 ± 0.76	0.9157	>0.9999	>0.9999

RMP: resting membrane potential; AP: action potential; fAHP: fast afterhyperpolarization potential. The number in parenthesis represents the number of neurons. p values of treatment were obtained from One-way ANOVA, p values between groups were obtained from Bonferroni post hoc comparison.

Supplementary Table 2. Membrane and action potential properties of BLA→NAc PNs

**Membrane properties**	Unstressed control (n = 14)	1d P-FS (n = 13)	7d P-FS (n = 13)	$p_{\text{treatment}}$	$p_{\text{1d P-FS vs. Unstressed control}}$	$p_{\text{7d P-FS vs. Unstressed control}}$
RMP (mV)	-65.10 ± 0.42	-64.62 ± 1.12	-63.51 ± 1.04	0.1865	>0.9999	0.1657
Input resistance (MΩ)	128.06 ± 5.95	122.47 ± 5.27	129.34 ± 7.19	0.7120	>0.9999	>0.9999
AP threshold (mV)	-28.84 ± 1.03	-30.29 ± 0.74	-29.44 ± 0.98	0.5490	0.5560	> 0.9999
Half width of AP (ms)	1.34 ± 0.06	1.45 ±0.04	1.33 ± 0.06	0.2382	0.3264	>0.9999
Amplitude of AP (mV)	76.50 ± 1.47	77.85 ± 1.43	76.85 ± 1.09	0.7650	0.9625	>0.9999
fAHP (mV)	-5.07 ± 0.76	-6.59 ± 0.82	-5.75 ± 0.67	0.3681	0.3210	>0.9999

RMP: resting membrane potential; AP: action potential; fAHP: fast afterhyperpolarization potential. The number in parenthesis represents the number of neurons. p values of treatment were obtained from One-way ANOVA, p values between groups were obtained from Bonferroni post hoc comparison.
