# Supplementary material for: Sgk1 upregulation in hippocampus-projecting amygdala neurons underlies the delayed onset of PTSD-like avoidance behavior
Source: Nat Commun. 2026 Apr 1;17:4683. doi: 10.1038/s41467-026-71129-0 (PMC13201608; doi:10.1038/s41467-026-71129-0)
Supplement: Supplementary file 2 — Description of Additional Supplementary Files [file 41467_2026_71129_MOESM2_ESM.pdf]

Description of Additional Supplementary Files

Supplementary Data 1. Statistical analyses related to Figures 1-7 and Supplementary Figures 1-20.

Supplementary Data 2. Primers used in this study.
