# Supplementary material for: Sgk1 upregulation in hippocampus-projecting amygdala neurons underlies the delayed onset of PTSD-like avoidance behavior
Source: Nat Commun. 2026 Apr 1;17:4683. doi: 10.1038/s41467-026-71129-0 (PMC13201608; doi:10.1038/s41467-026-71129-0)
Supplement: Supplementary file 5 — Reporting Summary [file 41467_2026_71129_MOESM5_ESM.pdf]

nature portfolio

Corresponding author(s): Bing-Xing Pan

Last updated by author(s): Feb 4, 2026

Reporting Summary

Nature Portfolio wishes to improve the reproducibility of the work that we publish. This form provides structure for consistency and transparency in reporting. For further information on Nature Portfolio policies, see our Editorial Policies and the Editorial Policy Checklist.

Statistics

For all statistical analyses, confirm that the following items are present in the figure legend, table legend, main text, or Methods section.

n/a	Confirmed	
☐	☒	The exact sample size (*n*) for each experimental group/condition, given as a discrete number and unit of measurement
☐	☒	A statement on whether measurements were taken from distinct samples or whether the same sample was measured repeatedly
☐	☒	The statistical test(s) used AND whether they are one- or two-sided<br>*Only common tests should be described solely by name; describe more complex techniques in the Methods section.*
☐	☒	A description of all covariates tested
☐	☒	A description of any assumptions or corrections, such as tests of normality and adjustment for multiple comparisons
☐	☒	A full description of the statistical parameters including central tendency (e.g. means) or other basic estimates (e.g. regression coefficient) AND variation (e.g. standard deviation) or associated estimates of uncertainty (e.g. confidence intervals)
☐	☒	For null hypothesis testing, the test statistic (e.g. *F*, *t*, *r*) with confidence intervals, effect sizes, degrees of freedom and *P* value noted<br>*Give P values as exact values whenever suitable.*
☒	☐	For Bayesian analysis, information on the choice of priors and Markov chain Monte Carlo settings
☒	☐	For hierarchical and complex designs, identification of the appropriate level for tests and full reporting of outcomes
☒	☐	Estimates of effect sizes (e.g. Cohen's *d*, Pearson's *r*), indicating how they were calculated

Our web collection on statistics for biologists contains articles on many of the points above.

Software and code

Policy information about availability of computer code

Data collection	Behavior data of open field test and elevated plus maze test were collected using the video-tracking system (Med Associates Inc., Fairfax, VT).<br>Behavior data of contextual fear conditioning test were collected using a the Video Freeze systerm (Med Associates Inc., Fairfax, VT).<br>Calcium signals were collected by the optical fiber recording system (Thinker Tech Nanjing Biotech Limited Co., Ltd), and the behavior tracking during recording were collected using a video camera (Softmaze, XR-Video).<br>Immunofluorescence images were taken by using a scanning laser scanning microscope (Nikon A1, Nikon, Japan).<br>Electrophysiological data were collected using Axon's Clampex software (Molecular Device, San Jose, CA, USA).<br>The optical density was measured with an EnSpire Multimode Plate Reader (PerkinElmer, USA).<br>Data of RT-qPCR were acquired using Step OnePlusTM software (Thermo Fisher Scientific, Waltham, USA).
Data analysis	Behavior data were analyzed using ANY-maze software (Stoelting Co., Wood Dale, USA).<br>Calcium signals were analyzed using MATLAB (The MathWorks, Natick, USA).<br>Imaging data were analyzed using Image J software (NIH, Bethesda, MD, USA).<br>Electrophysiological data were analyzed using Clampfit software 11.2 (Molecular Device, San Jose, CA, USA).<br>Statistical analysis were performed by GraphPad Prism 9 (GraphPad Software, La Jolla, USA) and SPSS 27.0 (IBM, Armonk, USA).

For manuscripts utilizing custom algorithms or software that are central to the research but not yet described in published literature, software must be made available to editors and reviewers. We strongly encourage code deposition in a community repository (e.g. GitHub). See the Nature Portfolio guidelines for submitting code & software for further information.

April 2023

Data

Policy information about availability of data

All manuscripts must include a data availability statement. This statement should provide the following information, where applicable:

- Accession codes, unique identifiers, or web links for publicly available datasets
- A description of any restrictions on data availability
- For clinical datasets or third party data, please ensure that the statement adheres to our policy

All data generated and analyzed during this study are included in this article and its supplementary information files. Source data are provided with the paper.

Research involving human participants, their data, or biological material

Policy information about studies with human participants or human data. See also policy information about sex, gender (identity/presentation), and sexual orientation and race, ethnicity and racism.

Reporting on sex and gender	N/A
Reporting on race, ethnicity, or other socially relevant groupings	N/A
Population characteristics	N/A
Recruitment	N/A
Ethics oversight	N/A

Note that full information on the approval of the study protocol must also be provided in the manuscript.

Field-specific reporting

Please select the one below that is the best fit for your research. If you are not sure, read the appropriate sections before making your selection.

☒ Life sciences ☐ Behavioural & social sciences ☐ Ecological, evolutionary & environmental sciences

For a reference copy of the document with all sections, see nature.com/documents/nr-reporting-summary-flat.pdf

Life sciences study design

All studies must disclose on these points even when the disclosure is negative.

Sample size	Sample sizes were determined based on previous experience and related literature. Sample sizes are indicated in the legends of each figure
Data exclusions	Pre-established exclusion criteria for experimental data points included lack of accurate stereotaxic targeting and/or viral expression, as verified by post-mortem brain tissue analysis. No outliers were excluded in this study.
Replication	All measurements were taken from distinct samples.
Randomization	The animals in the experiments were randomized assigned.
Blinding	For the physiological experiments, investigators were blinded to group allocation during data collection and analysis. The behavior data were collected and analyzed using computer software in an unbiased manner, rendering blinding unnecessary.

Reporting for specific materials, systems and methods

We require information from authors about some types of materials, experimental systems and methods used in many studies. Here, indicate whether each material, system or method listed is relevant to your study. If you are not sure if a list item applies to your research, read the appropriate section before selecting a response.

Materials & experimental systems

n/a	Involved in the study
☐	☒ Antibodies
☒	☐ Eukaryotic cell lines
☒	☐ Palaeontology and archaeology
☐	☒ Animals and other organisms
☒	☐ Clinical data
☒	☐ Dual use research of concern
☒	☐ Plants

Methods

n/a	Involved in the study
☒	☐ ChIP-seq
☒	☐ Flow cytometry
☒	☐ MRI-based neuroimaging

Antibodies

Antibodies used	Primary antibodies:<br>c-Fos antibody (synaptic systems, 226008, 1:3000)<br>SGK1 Polyclonal antibody (Proteintech, 23394-1-AP, 1:200)<br>mCherry Monoclonal Antibody (16D7) (Thermo Fisher, M11217, 1:500)<br>Anti-green fluorescent protein antibody (Aves Labs, GFP-1020, 1:500)<br>anti-pCREB Ser133 antibody (Upstate, 06-519, 1:800)<br>Fluorescent secondary antibody:<br>Donkey anti-rabbit Alexa Fluor 647 (Life Technology, A31573, 1:500)<br>Donkey anti-rabbit Alexa Fluor 488 (Thermo Fisher Scientific, A21206, 1:500)<br>Alexa Fluor 594 donkey anti rat (Thermo Fisher, A21209, 1:100)<br>Alexa Fluor 488 conjugated affinpure donkey anti chicken (Jackson ImmunoResearch, 703-545-155, 1:100).
Validation	All antibodies used in this study were obtained from commercial suppliers and were validated by the manufacturers. The validation is reported on their websites.

Animals and other research organisms

Policy information about studies involving animals; ARRIVE guidelines recommended for reporting animal research, and Sex and Gender in Research

Laboratory animals	Male and female C57BL/6J mice (4-10 weeks) were used for all experiments.<br>Virus injection and cannula implantation were performed on mice at 4-5 weeks of age.<br>Beads were injected at 6 weeks of age.<br>Behavioral tests, histological analysis, slice electrophysiological and fiber photometry were conducted around 10 weeks of age.
Wild animals	The study did not involve wild animals.
Reporting on sex	Both male and female mice were used in this study.
Field-collected samples	This study did not involve samples collected from the field.
Ethics oversight	All experimental procedures were under the National Institutions of Health guidelines and were approved by the Institutional Animal Care and Use Committee of Nanchang University (NCULAE-20221228059).

Note that full information on the approval of the study protocol must also be provided in the manuscript.

Plants

Seed stocks	N/A
Novel plant genotypes	N/A
Authentication	N/A
